# The development of a web- and a print-based decision aid for prostate cancer screening

**DOI:** 10.1186/1472-6947-10-12

**Published:** 2010-03-03

**Authors:** Caroline S Dorfman, Randi M Williams, Elisabeth C Kassan, Sara N Red, David L Dawson, William Tuong, Elizabeth R Parker, Janet Ohene-Frempong, Kimberly M Davis, Alexander H Krist, Steven H Woolf, Marc D Schwartz, Mary B Fishman, Carmella Cole, Kathryn L Taylor

**Affiliations:** 1 Department of Oncology, Lombardi Comprehensive Cancer Center, Georgetown University Medical Center, 3300 Whitehaven Street, NW Suite 4100, Washington, DC 20007-2401, USA; 2 The Clear Language Group, 7907 Roneale Drive, Elkins Park, PA 19012-1115, USA; 3 Department of Family Medicine, Virginia Commonwealth University, PO Box 980251 Richmond VA 23298-0251, USA; 4 Division of General Internal Medicine, Georgetown University Hospital, 3800 Reservoir Rd NW, Washington, DC 20007, USA; 5 Division of General Internal Medicine, Washington Hospital Center, 110 Irving Street, NW, Washington, DC 20010, USA

## Abstract

**Background:**

Whether early detection and treatment of prostate cancer (PCa) will reduce disease-related mortality remains uncertain. As a result, tools are needed to facilitate informed decision making. While there have been several decision aids (DAs) developed and tested, very few have included an exercise to help men clarify their values and preferences about PCa screening. Further, only one DA has utilized an interactive web-based format, which allows for an expansion and customization of the material. We describe the development of two DAs, a booklet and an interactive website, each with a values clarification component and designed for use in diverse settings.

**Methods:**

We conducted two feasibility studies to assess men's (45-70 years) Internet access and their willingness to use a web- vs. a print-based tool. The booklet was adapted from two previous versions evaluated in randomized controlled trials (RCTs) and the website was created to closely match the content of the revised booklet. Usability testing was conducted to obtain feedback regarding draft versions of the materials. The tools were also reviewed by a plain language expert and the interdisciplinary research team. Feedback on the content and presentation led to iterative modifications of the tools.

**Results:**

The feasibility studies confirmed that the Internet was a viable medium, as the majority of men used a computer, had access to the Internet, and Internet use increased over time. Feedback from the usability testing on the length, presentation, and content of the materials was incorporated into the final versions of the booklet and website. Both the feasibility studies and the usability testing highlighted the need to address men's informed decision making regarding screening.

**Conclusions:**

Informed decision making for PCa screening is crucial at present and may be important for some time, particularly if a definitive recommendation either for or against screening does not emerge from ongoing prostate cancer screening trials. We have detailed our efforts at developing print- and web-based DAs to assist men in determining how to best meet their PCa screening preferences. Following completion of our ongoing RCT designed to test these materials, our goal will be to develop a dissemination project for the more effective tool.

**Trial Registration:**

NCT00623090

## Background

Prostate cancer (PCa) is the leading cancer diagnosis among men and the second leading cause of male cancer death [1,2]. While research has shown that PCa screening can find cancer at its earliest stages, it is uncertain whether early detection and treatment of PCa leads to a reduction in disease-related mortality [3,4]. Preliminary results from two large randomized controlled cancer screening trials (RCTs) have recently been published [5,6]. While one trial found a 20% reduction in death from PCa as a result of screening [6], findings from the other trial showed no significant reduction in disease-related mortality [5]. Given these inconclusive results, the uncertainties regarding screening continue. The final mortality results from these trials will not be available for several years.

The challenge of making medical decisions prior to the availability of definitive outcome data has been a long-standing issue in cancer screening [7-9] that is likely to become increasingly important as advances in screening technology outpace our ability to validate effectiveness [10-14]. Currently, there is no national standard of care with regard to PCa screening and national medical organizations differ in their screening recommendations [15-21]. However, most recommend that men learn about the pros and cons of PCa screening in order to reach an informed decision [16-19,21]. Thus, widely applicable and easily disseminable approaches to health education are needed [22].

Informed decision making occurs when individuals understand the benefits, risks, alternatives and uncertainties surrounding a medical condition or procedure and are able to use this information in conjunction with their preferences to make a decision that is consistent with those preferences [23]. One approach to promoting informed decisions for PCa screening is the use of patient education materials and decision aids (DAs). The goals of DAs are to foster informed health decisions by: 1) providing facts about the condition and procedures; 2) helping patients to clarify personal preferences and values; and 3) encouraging discussions with medical professionals to guide health decisions that match these preferences [24]. DAs are considered particularly useful when efficacy is unclear, outcomes are uncertain, and/or subjective judgments about benefits and risks are required [24]. Cancer screening-related DAs are particularly important because they have been found to increase cancer-related knowledge without increasing anxiety [25].

Increasingly, cancer screening decision tools have begun to utilize the Internet [26-31]. Despite the digital divide and differences in Internet use among different age and racial groups [32], the percentage of Americans who use the Internet is continuing to grow [33,34]. Internet users who access medical or health information have also increased between 2001 and 2007, from 66% to 76% for those aged 50 to 64 and from 60% to 71% for those over 65 [32]. These trends suggest that the Internet has vast potential as a widely accessible approach to delivering decision support materials for PCa screening.

The most recent systematic review of DAs for PCa screening was published in 2007, which presented the findings of 12 RCTs [35]. While we did not conduct our own systematic review, we used the same Medline search criteria used in Volk's review ('prostate cancer' and 'decision making') to locate RCTs published since the review, between January 2007 and June 2009. We located an additional six trials [27-29,36-38], and thus there have been a total of 18 published RCTs evaluating materials designed to improve informed decision making regarding PCa screening [26-29,36-49]. These trials have assessed print, verbal, Internet, video and interactive computer-based PCa screening DAs. We have provided a summary of these 18 published RCTs (see Additional File 1). Quasi-experimental studies [50-54], abstracts, and studies evaluating DAs designed to increase PCa screening were excluded from our summary. Of the 16 trials that assessed knowledge, all reported a significant improvement. However, inconsistencies were seen among the trials with respect to changes in decisional conflict, screening behavior, intent to screen, and active participation in the screening decision (see Additional File 1).

Although these were well-conducted trials, there were several limitations in the development and evaluation of these DAs. First, only 5 of the 18 studies [27,38,40-42] included a values clarification component to assist men in integrating the information and elucidating their preferences about PCa screening. This may explain why most studies reported only a modest improvement in participants' knowledge, or modest reductions in decisional conflict [27-29,37,40,41,43-46,48]. Second, of the 4 web-based DAs, only one [27] utilized an interactive format, while the other studies with web-based interventions did not exploit the strengths of this medium [26,28,29]. Third, while one web-based tool utilized a tracking mechanism to monitor whether participants viewed the website [27], that study did not determine whether the amount of time spent and topics accessed on the site impacted outcome measures. Fourth, several of the studies reporting pre- and post-intervention evaluations had a brief follow up period of less than 1 month, thereby limiting the understanding of the long-term impact of the interventions on screening behavior and other outcomes [28,38,40,41,43,44]. Finally, only 4 of the RCTs included a substantial number of African American (AA) men, who are at greatest risk for PCa [36,38,42,45].

Our goal was to extend these prior studies by developing two new patient DAs, a booklet and an interactive web-based tool, that could be utilized in a variety of settings. We sought to create widely disseminable and relevant materials that would improve PCa knowledge and assist a heterogeneous population of men in making informed screening decisions. We incorporated a values clarification component into each DA, which is intended to help individuals determine their personal preferences and beliefs about PCa screening and to make informed choices in accordance with those preferences. We are currently conducting a three arm RCT (including a usual care arm) to assess the efficacy of these tools among a diverse sample of men accrued from primary care clinics. This paper describes the development and content of these two DAs, including a description of our prior feasibility studies and randomized trials, each of which contributed to the evolution of these tools.

## Methods

### Development of Decision Aids

The development of the booklet and website was guided by two print booklets we had previously created and evaluated (Studies 1 and 2). Further, we conducted two feasibility studies and usability testing to confirm the viability of developing and testing a web-based decision tool (Study 3). All studies were approved by the Georgetown University/Medstar Oncology Institutional Review Board.

#### Study 1- The Right Decision is Yours: A Guide to Prostate Cancer Check-ups

Our initial version of the booklet was developed in collaboration with the Most Worshipful Prince Hall Grand Lodge of the District of Columbia (Masons) and was targeted to AA men and their spouses [55]. We conducted eight focus groups (N = 44) with AA men between the ages of 40 and 70 to determine the target population's informational needs and to guide the content and format of the booklet. We conducted two additional focus groups with internists, family physicians, and urologists to obtain input about factual information to include in the booklet.

Thematic analyses of transcripts of the lay focus groups, along with input from the physicians and guidelines of the Centers for Disease Control and Prevention for the development of educational materials (i.e., clear presentation, logical sequence, ease in understanding, and interesting, familiar, realistic, positive images) aided in the creation of pilot materials [55,56]. We modified the pilot materials based on iterative feedback from focus groups, members of the Prince Hall Masons, and the entire project team.

The end product, completed in May 2000, was a 16-page educational booklet entitled *The Right Decision is Yours: A Guide to Prostate Cancer Check-ups *and targeted specifically to AA men [55,57]. We found that the print intervention increased knowledge and reduced decisional conflict when compared to videotape and control conditions, and that screening behavior was not associated with either of the interventions [45].

#### Study 2- Prostate Cancer Screening: Making an Informed Decision

In our next study, we revised the above booklet to target men of all ethnic and racial backgrounds. The additions to the content were adapted from a Centers for Disease Control and Prevention educational tool [58] and provided information about the leading causes of death among men, the accuracy of the prostate-specific antigen (PSA) test, and the treatment decisions that need to be considered when PCa is diagnosed.

Additionally, we included a 10-item values clarification component (adapted from Gattellari and Ward, 2003) [40] to help participants weigh the relative benefits and risks of screening. The balance sheet consisted of five items that addressed the benefits of screening (e.g., "I am worried about PCa and screening may give me peace of mind") and five items that addressed the limitations of screening (e.g., "I do not want to risk finding out I have cancer when it may never bother me"). Participants were asked to consider each item and indicate those 'that sound like you.' The purpose of the exercise was to provide men with a descriptive rather than a prescriptive summary of the screening objectives that were important to them. Their response patterns suggested whether they were leaning toward or away from getting screened.

We conducted 8 usability testing sessions with 3-4 men per session (total N = 29; 74% AA, 22% White, and 4% of Caribbean/West Indian descent; age 40-70). The majority of participants were recruited from fliers posted at Georgetown University Hospital (GUH), Howard University Hospital, the National Prostate Cancer Coalition, and the local fire department. Participants provided feedback on the style and method of presentation of the information to ensure that the booklet addressed relevant topics and that the uncertainty surrounding screening was addressed in a balanced manner. Further, participants gave their opinion of the values clarification component and suggested ways to improve the balance sheet.

Based on the usability testing findings and research team recommendations, a plain language specialist was consulted to ensure that the DA did not exceed an 8^th ^grade reading level. The end product, a 24 page booklet entitled *Prostate Cancer Screening: Making an Informed Decision*, was completed in July, 2004. We evaluated the booklet in a RCT among men who were registered to undergo free screening [59], comparing this booklet to the PSA question and answer fact sheet developed by the National Cancer Institute [60]. Similar to Study 1, exposure to the DA resulted in a significant increase in knowledge and a decrease in decisional conflict but no change in screening behavior.

#### Study 3- Prostate Cancer Screening: Making the Best Choice

##### Overview

The development of our final set of materials was conducted in several steps. We conducted two studies to assess the feasibility of an Internet-based PCa screening decision tool. Next, we drafted both the new booklet and the website based on the materials described in Studies 1 and 2. Finally, we conducted usability testing to obtain feedback on our draft materials.

##### Feasibility Studies

We sought to gain an understanding of our target population's access to and knowledge of the Internet. To do this, we conducted two feasibility studies with men accrued from the primary care clinics at two Washington, DC teaching hospitals, GUH and the Washington Hospital Center (WHC), the accrual sites for the target population in the ongoing RCT. GUH and WHC serve different populations with regard to race/ethnicity and socioeconomic backgrounds. GUH serves a racially diverse and middle to upper-middle class patient population, while WHC serves a largely AA patient population of predominately lower- to middle-class socioeconomic status.

We conducted feasibility studies with patients from both hospitals to determine how our materials should be tailored in order to meet the needs of each group. The feasibility studies were conducted 18 months apart to examine how Internet access and use changed among this population of men over time (January 2005 and June 2006). For each study, men between the ages of 45 and 70 without a previous diagnosis of PCa were accrued from the waiting rooms of the primary care clinics at GUH and WHC. Participants completed a brief survey that contained questions about sociodemographic information, PCa screening knowledge, and typical Internet use (see Table 1 for the sample description) (See Additional file 2). In the second study, we added the Newest Vital Sign component, a measure of medical literacy, to the questionnaire (See Additional file 3) [61].

**Table 1:** Feasibility Study 1, January 2005

	GUH (N = 34)	WHC (N = 21)	Total (N = 55)
**Age (N = 55)**	M = 55.4 SD = 8.2	M = 53.7 SD = 6.7	M = 54.7 SD = 7.6
			
**Race/Ethnicity #**			
White (N = 19)	50%	10.5%	35.8%
African American (N = 30)	38.2%	89.5%	56.6%
Other (N = 4)	11.8%	0	7.5%
			
**Education**			
< HS grad (N = 14)	20.6%	33.3%	25.5%
Voc/trade or some college (N = 12)	14.7%	33.3%	21.8%
College Graduate (N = 7)	20.6%	0	12.7%
Graduate work/degree (N = 22)	44.1%	33.3%	40.0%
			
**Marital Status**			
Married (N = 37)	70.6%	61.9%	67.3%
Other (N = 18)	29.4%	38.1%	32.7%
			
**Internet access at home/work**			
Yes (N = 41)	82.4%	61.9%	74.5%
No (N = 14)	17.6%	38.1%	25.5%
			
**Home/Work Computer Internet Usage **(among those with access at home/work)			
Few times yr/few times month (N = 7)	7.1%	38.5%	17.1%
Once/twice a wk (N = 5)	14.3%	7.7%	12.2%
Daily (N = 29)	78.6%	53.8%	70.7%
			
**Receiving Health Related Information #**			
Prefers Internet (N = 23)	47.1%	36.8%	43.4%
Prefers Booklet (N = 30)	52.9%	63.2%	56.6%
			
**Willingness to Read Prostate Cancer Info on the Internet #**			
Definitely/Probably would (N = 42)	76.5%	84.2%	79.3%
Definitely/Probably would not (N = 11)	23.5%	15.8%	20.7%
			
**Awareness of Disagreement in Medical Community Regarding Whether to Screen for PrCa**			
Unaware of disagreement (N = 45)	82.4%	81.0%	81.8%
Aware of disagreement (N = 4)	2.9%	14.3%	7.3%
Not Sure (N = 6)	14.7%	4.8%	10.9%

# N = 2 subjects with missing data

GUH = Georgetown University Hospital, WHC = Washington Hospital Center

##### Development of the Booklet and the Website

The study team and consultants first drafted a new version of the booklet. The primary differences from the prior booklet included both substantive changes (e.g., additional information on screening recommendations, different methods of PSA measurement, and additional figures and statistics) as well as improved readability (e.g., layout changes, improved wording, adding text boxes to highlight main points, greater use of bulleted text, reordering of topics). At each step in the development process, members of our research team, including primary care physicians and researchers specializing in PCa screening education, reviewed and modified drafts of the booklet.

After finalizing the content of the new booklet, we began working with the web developers to design the website prototype. We provided them with both the draft form of the booklet and a list of website features to include or avoid, based on our review of several existing health websites. We sought to create a universally functional site by accommodating varying web-browsers (e.g., Internet Explorer, Safari), using Adobe Flash ™ in the user interface, and using open source development tools to facilitate flexible site maintenance and support. The time required to load pages made the website unsuitable for dial-up connections. However, the results of the second feasibility study confirmed widespread access to high-speed Internet, and we anticipated even greater broadband use after the completion of the randomized trial.

The booklet and website were edited by a plain language expert who provided guidance on the presentation of the information, including the use of parallel sentence construction, bolded headers and sub-headers to alert readers to changes in topic, the use of bulleted text and tabs on the right edge of the pages, and the inclusion of a detailed glossary that defined medical terms often misunderstood by laypersons. The presentation of content on the website was designed to improve its appeal to persons who may not be regular web-users. We omitted sections of text to increase readability and wrote the materials in the conditional tense to prevent the reader from misinterpreting the information. Importantly, we acknowledged men's uncertainty surrounding screening in an effort to help them consider information that conflicted with their prior beliefs. Both the booklet and the website were written at or below an 8^th ^grade reading level based on the Fleish-Kincaid grade level formula [62].

The development of these materials was also guided by criteria from the International Patient Decision Aid Standards (IPDAS) Collaboration, a worldwide group of health-care practitioners and researchers who have developed standards for DAs [63]. IPDAS standards help researchers create DAs to prepare patients to have conversations with their physicians about medical tests and procedures [64].

##### Booklet Usability testing

We recruited participants (N = 14) from GUH and WHC primary care clinic waiting rooms, from fliers placed in surrounding neighborhoods, and from a General Education Development center to ensure inclusion of men with limited literacy. Men were eligible if they were 45-70 years old and had not had PCa. Participants reviewed the booklet in our research offices (N = 6), as well as the clinic waiting room (N = 7) and their own home (with follow-up to discuss his feedback; N = 1) in order to accommodate their schedules.

At the start of each session, one to two members of the research team held a brief discussion with participants regarding their prior experiences with screening to ensure that no one had had unusual experiences that would impact their feedback. Participants then individually reviewed the booklet and completed a brief questionnaire concerning their opinions of the DA, their overall health, and demographic information (See Additional file 4). Moderators noted participants' recommendations for modifications to the text, graphs, figures, and their impressions of the overall message of the DA.

Our intention was not to conduct traditional qualitative analyses, as we had previously done in Study 1 [55]. The majority of the content was decided upon from the previous versions of our materials and through updates gained from the CDC [58]. We conducted the usability testing to assess men's reactions to the presentation of the materials. The usability testing concluded with a brief questionnaire that inquired about men's opinions of the materials and demographic information.

##### Website Usability testing

Participants for the usability testing were again recruited from GUH and WHC primary care clinics. Usability testing of the website involved the same recruitment method and protocol described above for the booklet usability testing. However, all but one session was conducted in our research offices. Following the consent process, we provided an explanation of the rationale for the website and then asked participants to individually review the proposed website content. As in the booklet usability testing, website usability testing sessions were not recorded. However, one to three members of the research team observed each participant during the review process to look for navigation and usability issues and take note of verbal comments made by participants. The meetings ended with an informal discussion and a questionnaire to assess participants' thoughts, likes, and dislikes of the materials (See Additional file 5).

## Results

### Feasibility Studies

#### Sample

For the initial feasibility study (January 2005), 55/58 (95%) men agreed to participate. The mean age of participants was 54.7 (SD = 7.6), with a little over half of the men reporting that they were AA (Table 1).

The second feasibility study (June 2006) had a participation rate of 83% (99/119). The sociodemographic characteristics of these participants were virtually identical to that of the first feasibility study (see Table 2), with a mean age of participants of 54.6 (SD = 7.4) and just over half AA.

**Table 2:** Feasibility Study 2, June 2006

	GUH (N = 50)	WHC (N = 49)	Total (N = 99)
**Age (N = 98) #**	M = 53.9 SD = 7.5	M = 55.2 SD = 7.3	M = 54.6 SD = 7.4
			
**Race/Ethnicity †**			
White (N = 35)	52.0%	20.0%	36.8%
African American (N = 48)	28.0%	75.6%	50.5%
Other (N = 12)	20.0%	4.4%	12.6%
			
**Education**			
< HS grad (N = 25)	10.0%	40.8%	25.3%
Voc/trade/some college (N = 19)	10.0%	28.6%	19.2%
College Graduate (N = 18)	26.0%	10.2%	18.2%
Graduate work/degree (N = 37)	54.0%	20.4%	37.4%
			
Marital Status *			
Married (N = 53)	78.0%	29.8%	54.6%
Other (N = 44)	22.0%	70.2%	45.4%
			
**Internet access at home/work**			
Yes (N = 70)	92.0%	49.0%	70.7%
No (N = 29)	8.0%	51.0%	29.3%
			
**Home/Work Computer Internet Usage **(among those with access at home/work)			
Never/Rarely (N = 1)	0.0%	4.2%	1.4%
Few times per year/few per month (N = 4)	2.2%	12.5%	5.7%
Once a week/several times a week (N = 14)	17.4	25.0%	20.0%
Daily (N = 51)	80.4%	58.3%	72.9%
			
**Receiving Health Related Information •**			
Prefers Internet (N = 45)	66.0%	30.4%	48.4%
Prefers Booklet (N = 43)	25.5%	67.4%	46.2%
No preference (N = 5)	8.5%	2.2%	5.4%
			
Willingness to go to another location if no access to high-speed Internet connection? *‡			
Yes (N = 16)	33.3%	45.2%	43.2%
No/Not sure (N = 21)	66.7%	54.8%	56.8%
			
**Newest Vital Sign (NVS)**^^ ^(N = 99)	M = 3.9 SD = 2.0	M = 1.9 SD = 1.8	M = 2.9 SD = 2.2

^#^N = 1 subject with missing data

† N = 4 subjects with missing data

* N = 2 subjects with missing data

• N = 6 subjects with missing data

‡ Includes participants with slow-speed Internet access and no Internet access

^^ ^Scores on the NVS range from 0 to 6, with fewer than four correct answers indicating the possibility of limited literacy

GUH = Georgetown University Hospital, WHC = Washington Hospital Center

#### Results

Responses to the first feasibility study indicated that, regarding PCa screening knowledge, 97.1% of men at GUH and 85.7% at WHC endorsed the belief that 'experts agree that all men should be tested for PCa.' This suggested a lack of understanding of the uncertainties surrounding screening. Overall, the majority of men with Internet access at home or work reported accessing the Internet a few times a week or daily (82.9%). Further, 79.3% of all men indicated they would 'probably' or 'definitely' read information about PCa on the Internet. We did not compare the two sites for statistically significant differences as the goal was simply to describe the men present at each site.

In the second feasibility study, a majority of men reported having Internet access at home or work (70.7%), and just over half reported preferring to receive health related information on the Internet (53.8%). The mean total score for the Newest Vital Sign scale was 3.9 (SD = 2.0) at GUH and 1.9 (SD = 1.8) at WHC. Scores on the Newest Vital Sign scale range from 0 to 6, with fewer than four correct answers indicating the possibility of limited literacy.

The feasibility studies identified the need for educational tools to improve men's knowledge of the uncertainties surrounding PCa screening. Responses to the Internet access and use questions confirmed that the Internet was a feasible medium for a widely accessible PCa screening educational tool. Although these were not representative samples, the data suggest that a majority of men had access to the Internet, and this access was sustained over time. These results further supported and gave us confidence in our decision to create a website that used a broadband Internet connection (as opposed to a dial-up connection) in order to deliver more complex interactive and video features. Participants' scores on the Newest Vital Sign reinforced the need for a plain language specialist in developing text for the website and the booklet.

### Booklet Usability testing

#### Sample

The mean age of participants (N = 14) was 53.8 (SD = 7.8). Half of the men were unemployed or retired and 71.4% were AA. Additional demographic information for these participants is presented in Table 3. Given the overlap between the groups of men who reviewed the booklet and the website (N = 6 reviewed both), we did not assess the potential differences between the two groups.

**Table 3:** Evaluation Data from Usability testing (Study 3)†

	Booklet	Web
	N = 14	N = 14
**Age**	M = 53.8	M = 54.0
	SD = 7.8	SD = 7.2
**Race**		
White	28.6%	50.0%
African American	71.4%	50.0%
**Education**		
**<**HS grad	21.4%	35.7%
Some college	42.9%	21.4%
College Graduate	14.3%	0.0%
Graduate work/degree	21.4%	42.9%
**Marital Status**		
Married/living as married	50.0%	35.7%
Not married	50.0%	64.2%
**Employment Status**		
Not employed/Retired	50.0%	50.0%
Employed	41.9%	50.0%
**Health Insurance**		
Yes	92.9%	78.6%
**How often Screened**		
3-6 months	7.1%	14.3%
Annually	50.0%	42.9%
Every 2 years	0.0%	0.0%
Don't know/Missing	42.9%	42.9%
**Prior Abnormal Screening Result**		
Yes	7.1%	7.1%
**Amount of Information Provided**		
Much/A little less info than wanted	7.1%	21.4%
About Right	50.0%	50.0%
A little more/a lot more info than wanted	42.8%	21.4%
**Length of booklet/website**		
Much too long/a little too long	35.7%	50.0%
About right	50.0%	42.9%
Wanted a little/much longer	14.2%	7.1%
**Clarity**		
Everything/most things clear	85.7%	92.9%
Some Clear	7.1%	7.1%
Many unclear	0.0%	0.0%
Missing data	7.2%	
**Overall Message**		
Definitely/Probably not screen	14.3%	14.3%
Neither	14.3%	35.7%
Definitely/Probably Screen	71.5%	42.8%

† No significance testing was conducted due to overlap between groups (6 men participating in web usability testing also participated in booklet usability testing)

#### Results

Responses to the questionnaire are presented in Table 3. Half of the men reviewing the booklet indicated that it had about the right amount of information and was about the right length, but a substantial minority reported that it contained more information than they would have liked (42.8%) or was too long (35.7%). Despite our efforts to provide balanced information, a majority of men (71.5%) said that the overall message of the booklet was that they should probably or definitely get screened for PCa. Many participants indicated that they were unaware of the uncertainty surrounding PCa screening. This suggested that a single discussion of the uncertainty was not enough to impact men's understandings of this complex topic.

**Figure 1:**
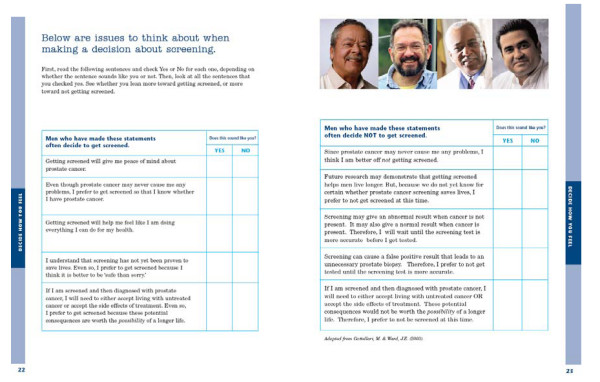
**Booklet Values Clarification Component (*Adapted from Gattellari & Ward (2003)***[40].

In general, men were positive about the booklet, but many provided suggestions for information they would like to have added (Table 4); the content and layout of the booklet were revised accordingly. For example, the values clarification component presented in the Study 2 booklet, containing a non-prescriptive balance sheet, was simplified based on feedback from usability testing participants (see Figure 1). Participants were asked to select which of 10 statements 'sound like you,' with five highlighting the benefits of screening and five highlighting limitations. Participants' responses were intended to indicate whether they leaned toward or away from screening. Because many men found the original questions to be complex, we tested multiple versions of the questions.

**Table 4:** Feedback and Subsequent Changes from Usability testing (Study 3)

Concern	Solution
Booklet Usability testing	

Questions about age- and race- adjusted PSA	- Sections added with this material

Complex material	-Plain language consultant was used
	-Changed in text based on comments from men
	-Used bullet points and short sentences

Complex DA questions	-Multiple versions of the DA questions were created and tested

Website Usability testing	

Little experience using the Internet	- Instructions and a troubleshooting packet provided to user
	- Instructions provided on the website

Complex material	- Used less text on each screen
	- Increased use of bullet points
	- Audio summarized what is on the page for the participant
	- New "vocabulary" words have a hyperlink to a pop-up with their definition

Figure of prostate looks "cartoon-like"	- Changed figure and other graphics to look more realistic

Some men commented that they would like to see audio on the pop-ups.	- Web developers and researchers decided that this would be too distracting; this suggestion was not implemented.
	- Audio was put on all main pages for consistency.

Table 5 summarizes the booklet content and discusses the similarities and differences between the booklet and the web-based DA. We maintained consistency between the content of the booklet and website but note differences related to the interactive features of the web-based tool.

**Table 5:** Comparison of the Booklet and Web-based Educational Tools (Study 3)

Section	Summary of Content	Features/Differences between Booklet and Web
Title page & introduction	-Why you should read the material	- The website included a tutorial on how to use the program and its interactive features, such as video testimonials, pop-outs, and animated diagrams.
	-Includes table of contents	- The website required participants to answer a question regarding their current beliefs about screening before they began reviewing the website. This question was followed by 2 tailored video testimonials.
		- The booklet table of contents was located on the second page, and the booklet included section tabs along the edges of the pages for easy access to specific topics.
		-The website table of contents was presented along the left side of each screen and allowed participants to select where to begin.

Know the basics about the prostate gland	-What is the prostate, types of prostate problems	- The first values clarification question was presented on the website.

Understand why there is no right or wrong choice about prostate cancer screening	-Definition of screening, description of screening tests, screening recommendations from national organizations	- Two values clarification questions were presented on the website.
	- Information about whether screening will help men	- Two video testimonials were presented on the website.

Learn the facts about prostate cancer screening	-Steps involved in screening	- Four values clarification questions were presented on the website.
	-Screening accuracy	-The website provided pop-out boxes with additional information about PSA testing (i.e. PSA velocity, race- and age- adjusted PSA, free vs. attached PSA).
	-Is screening right for you	-The booklet had a tree branch diagram describing screening accuracy, while the website had an animated diagram with text and audio.
		-The booklet provided testimonial quotes from men who believed that screening was helpful and who questioned whether getting screened was helpful.

Facts you should know if prostate cancer is found---treatment issues	-Deciding whether to treat prostate cancer: the risks of engaging in watchful waiting and the risks of treating the cancer.	- Three values clarification questions were presented on the website.
	-Information about Gleason Score, PIN, and over treatment	- Two video testimonials were presented on the website.
	-Treatment decisions and factors to consider	- The website included pop-outs discussing the side effects of active treatment for prostate cancer.
	- Types of active Treatment	
	-Information about late stage prostate cancer	
	-Side effects of treatment	

Steps you can take to make the best choice about whether to be screened for prostate cancer	-Know your risk factors: age, history, race, diet	- The website provided pop-outs with statistics about risk factors, symptoms, etc. (provided charts and graphs).
	-Learn the symptoms	- The booklet provided a space where men could write in questions they would like to ask their doctor about prostate cancer screening.
	-Talk with your doctor about screening---includes questions to consider when discussing screening with your doctor	- The website allowed for men to print out the questions to ask their doctor that have been provided and urged men to write down any additional questions they had.

Values Clarification Exercise	-Instructions for how to complete the worksheet	-The booklet presented the worksheet questions on two pages, separating statements from men who decided to get screened from men who have decided not to get screened.
	- The worksheet---includes 10 questions to determine if men are leaning towards or away from screening	- The website allowed men to review and change their answers to questions, and to view a results page with a balance beam diagram. The balance diagram showed participants if they leaned toward screening or against screening. Men could print a summary of their responses.
		- Values clarification questions were asked throughout the site and were located in sections corresponding to the content of the question.

Learn more about prostate cancer	-Charts and graphs about ways to measure PSA, disease incidence and mortality, concerns about active treatment for older men, and side effects from treatment	- The information that was seen in pop-ups earlier in the website was also available again at this point in the website, but the information was available in the booklet for the first time.

Additional sources for information about screening	-Glossary	-The website provided hyperlinks to the websites of organizations that could provide men with further information about prostate cancer.
	-References	
	-Contact information for organizations	

General features	- Audio vs. text	-Only the website allowed for audio.
	- Pop outs vs. text boxes	-The website featured pop-out boxes, while the booklet had text boxes highlighting important information.
	- Graphics	-There were more visual features and graphics on the website than on the booklet, due to the nature of the website's design.
	- Testimonials	- The website presented 8 video testimonials, while the booklet presented 2 testimonial quotations.

### Website Usability testing

#### Sample

Overall, 14 men reviewed the website, six of whom had previously reviewed the booklet. The mean age of participants was 54.0 (SD = 7.2). Despite the small sample size, half of the participants were AA and half were employed. Additional demographic information for these participants is presented in Table 3.

#### Results

Based on the questionnaire data, one-half (50%) of the men said the site provided about the right amount of information and 42.9% said it was about the right length. Despite our efforts to ensure a balanced presentation of the issues, only 35.7% indicated that the website's overall message neither favored nor opposed PCa screening. Although far short of our goals, this was an improvement over our prior decision tools and over the current booklet (see Table 3). Anecdotal information suggested that this viewpoint may be most prevalent among men who were screened regularly, as they may have taken note of the benefits of screening more so than the limitations. Table 4 describes revisions made to the website based on usability testing feedback. Changes related to content were made to both the website and booklet.

A primary concern during website development was whether men with little or no computer experience could successfully use the site. Based on difficulties experienced by three participants with little to no computer experience, several necessary modifications were made to the site to make it more user-friendly (Table 4). Difficulties in site navigation would have been overlooked if only computer savvy men had been sampled. While some men initially had trouble using the website, they were all ultimately able to successfully navigate the site and understand its content after viewing printed instructions.

Based on usability testing and recommendations from the research team, the web developers created several iterations of the website before a release candidate was created, approved, and launched. The website required 30-50 minutes to review and had a literacy level that did not exceed an eighth grade reading level. Importantly, the website aimed to present information in a balanced manner, neither encouraging nor discouraging screening. The web developers continue to provide ongoing technical support to ensure that team members and study participants do not encounter difficulties using the website.

Table 5 also provides details on the nine topic areas presented in the website as well as the booklet, including content features, similarities and differences with regard to the presentation of information, as well as a description of how general features differ between the two formats (e.g., audio vs. text, graphics). The final versions of the booklet and website, entitled *Prostate Cancer Screening: Making the Best Choice*, were completed in October 2007. The remainder of the Results section is devoted to the description of specific interactive features of the website.

#### Components of the Website

The interactive features of the website enabled an expansion and customization of the material. For example, web-users could access additional external information (e.g., the resource page included links to the homepages of national organizations that provide more information on PCa screening and treatments) or could use the table of contents to easily navigate to other sections of interest.

The website was made more accessible to men with limited literacy by presenting the majority of the text via voice over. Audio was available for approximately 70% of the text, and 80% of the audio matched the text verbatim. Users could deactivate the audio if they preferred to only read text.

The website included eight testimonials, prepresented as video clips of men speaking about their screening decision, whereas the booklet included written quotations from two men (one pro, one con). The videos enabled users to view the emotional reactions and facial expressions of the different actors as they relayed personal stories about PCa screening. We utilized actors that represented a racially diverse group of men in an effort to help make the information more relevant and understandable [31]. Of the eight video testimonials presented, 3 of the actors were AA, two were white, one was Asian, one was Native American, and one was Hispanic.

The interactive nature of the website insured that users were exposed to a perspective about PCa screening that differed from their own, in an effort to address the presence of a confirmation bias [65]. To do this, users indicated their history of screening, which was assessed upon entry into the website. The first two video testimonials were tailored so that a user who was leaning *toward *screening first viewed two testimonials of men who chose *not *to get screened, and vice versa for men leaning against screening. In subsequent sections of the website, the remaining six testimonials (3 pro, 3 con) were presented back to back so that everyone viewed all 3 pairs. The race of the actors varied so that the pro and con messages were delivered by actors of different races.

**Figure 2:**
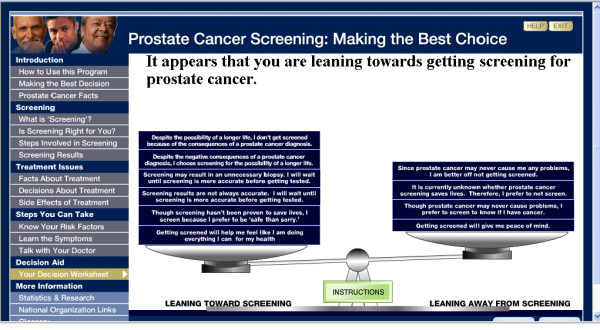
**Website Values Clarification Component (*Adapted from Gattellari & Ward (2003))***[40].

The 10-item values clarification component described above for the booklet was also included on the website in an interactive format (Figure 2). Individual items were presented at the end of different content sections and users were prompted to respond: 'sounds like me,' 'does not sound like me 'or 'not sure yet.' Questions that men skipped as they navigated through the site were automatically entered as 'not sure yet.' The questions were displayed again at the end of the website to provide men with an opportunity to change the response. Responses to all items were then depicted on a balance scale to help men determine if they leaned toward or away from getting screened. Finally, users always received a prompt to review the values clarification tool upon exiting the website; thus, it was seen by all men, even those who did not review the entire website.

**Figure 3:**
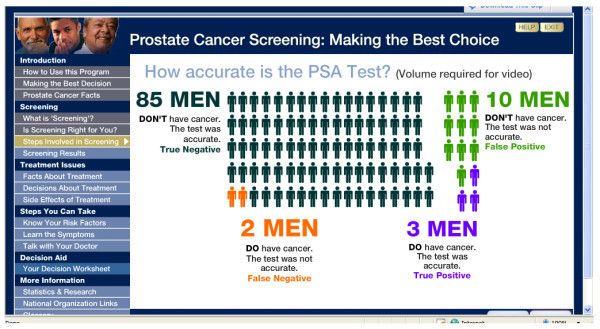
**Website Screenshot of Animation Depicting the Accuracy of the PSA Test **[83-85].

**Figure 4:**
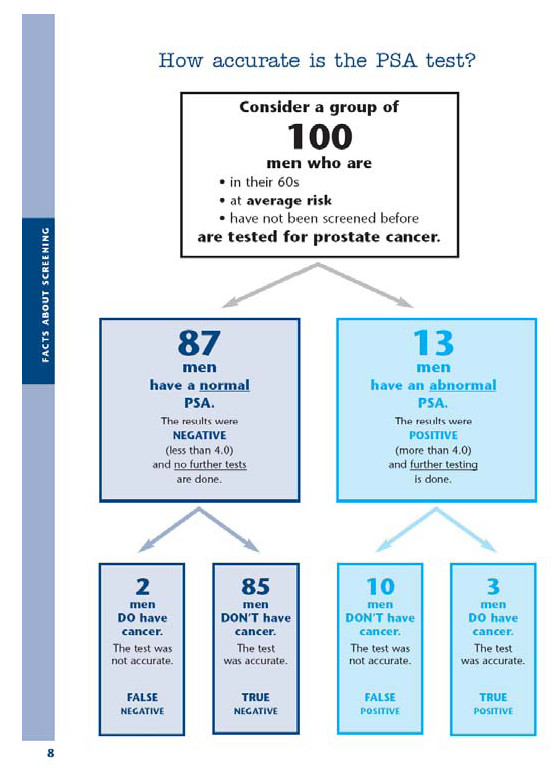
**Booklet Depiction of the Accuracy of the PSA Test **[83-85].

The web-based format also allowed for animation and graphics to draw attention to key points and direct the user through the website. For example, a graphic of 100 men that illustrated the accuracy of the PSA test changed colors to distinguish subgroups from the whole and was accompanied by a voiceover that explained the figure (Figure 3). The booklet, however, provided a single tree diagram to depict the same statistics (Figure 4).

Finally, the website is capable of tracking men's use of the materials and provides data on the behaviors of visitors. Data stored in a password protected Structured Query Language database provides records of the time spent visiting the website, time spent in each section, and responses to the DA queries. Upon completion of the RCT, this information will help determine whether initial screening preferences and usage patterns are associated with knowledge acquisition, decisional conflict, baseline screening preference, and subsequent screening decisions.

## Discussion

There is considerable interest in assisting men with PCa screening decisions, as evidenced by the 18 RCTs conducted to assess the efficacy of DAs. We sought to address several limitations of the previous studies with the development of two disseminable tools with extensive formative evaluations: a print booklet and an interactive website. The booklet and the website offer the identical content, while the website also includes an interactive values clarification component, video testimonials, and tracking software to assess men's utilization of the website. The website recently received the 2009 American Public Health Association's award for Public Health Education Materials [66]. Our ongoing RCT will examine the impact of the website and booklet on PCa screening knowledge, decisional conflict, satisfaction with decision, health-related quality of life and screening behaviors. The trial will be completed in 2011.

Our new materials offer several improvements over prior DAs. Given the persistent problem created by the digital divide, regarding both limited access and preferentially attracting Internet-savvy users, we designed features of the website to appeal to more diverse groups. Our formative work and prior randomized trials included a large number of AA participants as well as participants from diverse socioeconomic backgrounds, which provided insights to design materials for these groups. For example, our DAs maintained a maximum of an 8^th ^grade reading level, information was provided in plain language, and a glossary defined terms used throughout the website and booklet. Further, we incorporated visuals, graphs and charts, all of which have been suggested as methods for increasing comprehension among low-literacy groups [31].

In addition to making the materials appealing and understandable to diverse groups of men, we also assessed the extent of web access within our target sample. We found that over an 18-month period, web usage among lower socioeconomic men had increased. These results are consonant with some findings from the Pew Foundation [67], although other studies have reported slower growth among low SES and minority groups [68]. Our ongoing trial will provide more definitive evidence regarding the impact of web- vs. print-based media for PCa screening education, as well as the extent to which we managed to bridge the digital divide. The testing of these DAs among minorities and low literacy participants will provide an important opportunity to validate effectiveness in this population, which tends to be less informed about screening options and less engaged in decision-making [69,70].

To our knowledge, ours is the second study to describe an *interactive *web-based tool designed to educate men about PCa screening [27], and the first to utilize tracking software to compare website utilization patterns with patient outcomes. Potential advantages of using web-based materials include the relatively low cost of updating information and the increased capability of tailoring and interactivity, which may assist in the acquisition and integration of knowledge. For example, by tailoring the presentation of the first two video testimonials in response to the user's baseline screening preference, we have insured that men consider counterarguments to help balance their perspective. Also, the interactive nature of the web-based values clarification exercise was designed to actively engage participants and enhance the integration of knowledge. This was intended to help men make a decision that corresponds more closely with their own values and screening preferences, decrease decisional conflict, and increase decisional satisfaction.

Over the course of the development of these DAs, we have encountered several important issues that may be useful to others who are creating similar tools. First, there was a tradeoff between providing detailed information vs. risking that the materials would require more time than men would be willing to devote to them. Despite our best efforts to be concise, between 35% (booklet) and 50% (website) of the usability testing participants thought the materials were too long. As we did not want to exclude any pertinent information, it is possible that the length of our materials may deter some men from reading all of the text. However, with both the booklet and the website, we expect that men will selectively access sections of interest by using the Table of Contents.

Secondly, when we conducted our web usability testing, we provided a step-by-step instruction sheet for using the website. Men reported that the instructions were very helpful, particularly for those with less computer experience. Due to these findings, as well as what we know about the disparities between SES groups and Internet use, we have included the instruction sheet in the current randomized trial to ensure that men with less experience using the Internet will be able to successfully access and use our materials.

Finally, it was challenging to develop a DA that addressed the uncertainty of a screening test for men who had been undergoing regular screening *and *who were completely unaware of the uncertainty. The balancing act was to validate what men already knew while also providing information that was both counterintuitive and contrary to their previous impression. We worked to present the materials in an evenhanded fashion; however, results from the usability testing suggested that only a minority of men thought the booklet (14%) and website (36%) neither endorsed nor opposed screening. These findings highlight the fact that it may take more than a single exposure to materials such as ours for patients to grasp a message that is both complex and counter to one's current understanding and practices. While our usability testing provided critical information during the development process and our sample size was comparable to other studies involving usability testing [71,72], a larger sample may have provided the feedback necessary to more effectively present the uncertainty and the message of neither endorsing nor opposing screening.

## Conclusion

Despite the uncertainty surrounding PCa screening, most primary care physicians routinely order the PSA test for men over 50, and some engage in unsupported practices, such as screening patients over age 75 and referring such men for biopsies when PSA values are elevated [73-75]. Due to the logistic constraints they face [76], many physicians administer PCa screening with little opportunity to discuss the test beforehand [77]. However, evidence indicates that many men would prefer to make a shared PCa screening decision in conjunction with their physicians [78-81]. Consequently, access to an effective DA in the primary care setting may promote shared decisions among large numbers of men in the decisive period before testing occurs. In 2005, 80% of males had at least one visit with an ambulatory care physician [82], suggesting that an intervention implemented in this setting could have a widespread impact.

Providing assistance for informed decision making for PCa screening may be important for some time, particularly if a definitive recommendation either for or against screening does not emerge from the ongoing screening trials. We have detailed our efforts at developing print- and interactive web-based DAs to assist men in determining whether they prefer to be screened or not. Given that technological advances in medical screening tests will continue to occur faster than clinical translational research can keep pace, we hope that insights from the development of our decision tools will be applicable as other screening dilemmas arise.

## Competing interests

The authors declare that they have no competing interests.

## Authors' contributions

CD took the lead role in the writing and editing of the manuscript, as well as in usability testing recruitment. RW administered the questionnaires for the first feasibility study and participated in drafting the manuscript. EK participated in drafting the manuscript and in data analysis. SR participated in drafting the manuscript, conducting usability testing, and data analysis. DD recruited participants, conducted usability testing, and participated in data analysis. WT participated in drafting the manuscript. EP recruited participants, conducted usability testing, and participated in data analysis. J O-F was the plain language consultant during the iterative development process and provided multiple edits to both the layout and the wording of the text. KD, AK, SW, and MS participated in drafting the manuscript and reviewing and editing the materials during the iterative process. MF provided access to the primary care clinic in which men were recruited for the feasibility studies and usability testing, as well as drafting the manuscript and reviewing the materials during the iterative process. CC provided access to the primary care clinic in which men were recruited for the feasibility studies and usability testing, as well as drafting the manuscript and reviewing the materials during the iterative process. KLT conceived the project, is the Principal Investigator of the NCI and DoD grants which have funded this project, edited and reviewed the materials during the iterative process, and participated in writing the manuscript. All authors read and approved the final manuscript.

## Pre-publication history

The pre-publication history for this paper can be accessed here:

http://www.biomedcentral.com/1472-6947/10/12/prepub

## Supplementary Material

Additional file 1 **Appendix A**. Outcomes of Randomized Controlled Trials of Prostate Cancer Screening Decision Aids Click here for file

Additional file 2 **Supp File 2**. Pilot Screening and Internet Usage Questionnaire-Feasibility Study 1 - January 2005 Click here for file

Additional file 3 **Supp File 3**. Pilot Screening and Internet Usage Questionnaire-Feasibility Study 2 - June 2006 Click here for file

Additional file 4 **Supp File 4**. Booklet Usability Testing Questionnaire Click here for file

Additional file 5 **Supp File 5**. Website Usability Testing Questionnaire Click here for file
