# Supplementary material for: The development of a web- and a print-based decision aid for prostate cancer screening
Source: BMC Med Inform Decis Mak. 2010 Mar 3;10:12. doi: 10.1186/1472-6947-10-12 (PMC2845091; doi:10.1186/1472-6947-10-12)
Supplement: Additional file 1 — Appendix A. Outcomes of Randomized Controlled Trials of Prostate Cancer Screening Decision Aids [file 1472-6947-10-12-S1.DOC]

**Appendix A. Outcomes of Randomized Controlled Trials of Prostate Cancer Screening Decision Aids**

| **Reference** | **Subjects *** | **Follow Up Time** | **Media Tested** | | | | | | **Outcomes**  **(all results significant)** | | | | |
| --- | --- | --- | --- | --- | --- | --- | --- | --- | --- | --- | --- | --- | --- |
|  |  |  | **Usual Care** | **Print** | **Video** | **Verbal** | **Computer/ Web** | **Values Clarification Component** | **Improvement**  **in Knowledge** | **Reduced Decisional Conflict** | **Changes in Screening Behavior** | **Changes in Intent to Screen** | **Increase in Active Participation/ Engagement after viewing decision aid?** |
| ^+^ Frosch 2008 [27] | N=611 men over 50 yrs, 86% white | Un-specified | X ^c^ |  |  |  | X | Yes | Intervention groups had higher knowledge | Yes | Reduced PSA in both groups | Not assessed | Not assessed |
| ^+^Stamatiou 2008 [37] | N=1500 Greek men, ages 50-86 | 24 months |  | X |  | X |  | No | Print group had higher knowledge scores | Not assessed | Not assessed | Not assessed | Not assessed |
| ^+^ Volk 2008  [38] | Setting 1: n=149, low literacy health clinic, ~72.5% AA  Setting 2: n=301, high literacy health clinic,  ~18.6% AA | 2 weeks |  | X  (plus Audio) |  |  | X | Yes | Both groups at both sites had improvements in knowledge; no difference between groups | Both groups at both sites reduced decisional conflict | Not assessed | Not assessed | Low literacy men given website had greater active participation than same men given audio booklet; no difference at high literacy setting |
| ^+^Ilic 2008 [28] | N=161Australian men, 45 ys old and older | 1 week |  | X | X |  | X | No | Knowledge improved equally among all groups | No difference between groups | Not assessed | Not assessed | No change |
| ^+^ Kripalani 2007 [36] | N=250 men aged 45-70, 90.4% AA | None | X ^a^ | X |  |  |  | No | Not assessed | Not assessed | Interventions led to an increase in screening behavior | Not assessed | Both intervention groups showed increase in active participation |
| ^+^ Krist 2007 [29] | N=497 men, aged 50-70, 91% white | None | X | X |  |  | X | No | Intervention groups had greater knowledge (post-test only) | No | Intervention groups less likely to be screened | Not assessed | Both intervention groups showed increase in active participation |
| Taylor 2006 [45] | N=286 AA men, ages 40-70 | 1 month, 12 months | X | X | X |  |  | No | Both intervention groups increased knowledge compared to usual care | Both intervention groups decreased decisional conflict compared to usual care | Increase in screening occurred, but was not associated with interventions | Not assessed | Not assessed |
| Watson 2006 [47 | N=990 English and Welsh men, ages 40-75 | None | X | X |  |  |  | No | Intervention had higher knowledge than control (post-test only) | Not assessed | Not assessed | No difference between groups | No difference between groups |
| Gattellari and Ward 2005 [41] | N=421Australian men aged 50-70 | At least 1 week | X ^a^ | X | X |  |  | Yes (Print group only) | All groups improved knowledge at follow-up; those in print group had scores higher than video and usual care groups | No (post-test only) | Not assessed | Intervention groups had a decrease in intent to screen | Intervention groups increase in active participation |
| Myers 2005 [42] | N=242 AA men, ages 40-69 | 6 months | X ^a^ | X  (plus Verbal Values Clarification Component) |  |  |  | Yes | Not assessed | Not assessed | No significant difference | Not assessed | Not assessed |
| Partin 2004 [43] | N=1152 men, ages 50 and older, 95% white | 2 weeks | X | X | X |  |  | No | Both interventions had improved knowledge compared to control group | Not assessed | Not assessed | Intervention groups less likely to intend to have PSA test | Not assessed |
| Frosch 2003 [26] | N=226 men, aged 50 yrs and older, 91% white | Un-specified |  |  | X |  | X | No | Both groups showed an increase; video had higher knowledge at post-test | Not assessed | Fewer PSA tests requested at follow-up | Not assessed | Not assessed |
| Gattellari and Ward 2003 [40] | N=214 Australian men aged 40-70 | 3 days | X ^a^ | X |  |  |  | Yes | Intervention group had higher knowledge scores at post-test than control | Yes | Not assessed | No difference between groups; PSA interest decreased for both | Not assessed |
| Volk 2003 [46] | N=160 men, aged 45-70, 66% white | 2 weeks, 12 months | X ^a^ |  | X |  |  | No | Intervention group showed an increase in knowledge at follow-up | Not assessed | Intervention group less likely to have PSA | Intervention group reduced intent to screen | Not assessed |
| Wilt 2001 [48] | N=342 men, aged 50 years and older, 90% white | None | X | X |  |  |  | No | Intervention group had higher knowledge scores at post-test | Not assessed | No change | Not assessed | Not assessed |
| Schapira 2000 [44] | N=257 men, aged 50-80, 92% white | 2 weeks | X ^a^ | X |  |  |  | No | Intervention group had higher knowledge scores at post-test | Not assessed | Not assessed | Not assessed | Not assessed |
| Davison 1999 [39] | N=100 Canadian men, aged 50-79 | Un-specified | X ^b^ | X  (plus Verbal) |  |  |  | No | Not assessed | Intervention had lower levels of decisional conflict | Not assessed | Not assessed | Intervention took a more active role in decision making |
| Wolf 1996 [49] | N=205 men, 50 yrs or older; no prior PSA test, ~37% nonwhite | None | X ^b^ |  |  | X |  | No | Not assessed | Not assessed | Not assessed | Control group had greater interest in screening | Not assessed |

* = in all studies, subjects had no history of PCa

^+^ = study not included in Volk 2007 review

X ^a^ = study used a basic print intervention as usual care

X ^b^= study used a basic verbal intervention as usual care

X ^c^ = study used a basic web intervention as usual
