# Supplementary material for: The development of a web- and a print-based decision aid for prostate cancer screening
Source: BMC Med Inform Decis Mak. 2010 Mar 3;10:12. doi: 10.1186/1472-6947-10-12 (PMC2845091; doi:10.1186/1472-6947-10-12)
Supplement: Additional file 2 — Supp File 2. Pilot Screening and Internet Usage Questionnaire-Feasibility Study 1 - January 2005 [file 1472-6947-10-12-S2.PDF]

Internet and Print-Based Education for Prostate Cancer Screening

You can help us to understand the best ways of educating men about prostate cancer screening by completing this questionnaire. We would like to make information on prostate cancer screening available on the Internet, but need your feedback about whether this is a good idea or not. Please take a moment to complete the following questions. Thank you very much for your time and participation in our project.

1. How often do you use the Internet? (please check one)

___ never/rarely ___ few times a year ___ once a month ___ few times/month ___ once-twice/week
___ daily

2. Where do you access the Internet? (check as many as apply)

___ home ___ work ___ other location (library, friend's computer, Internet cafe)
___ I don't access the Internet

3. When you use the Internet, what sorts of information do you usually look for? (check all that apply)

___General health-related information (e.g., my health, my family's health, medications, etc.)
___ Information about prostate cancer
___ News, sports, weather, entertainment information
___ Work-related information
___ Email access (e.g., AOL, Yahoo, MSN accounts)
___ Other: ____________________
___ I don't access the Internet

4. Would you prefer to receive health-related information on the Internet or as a booklet? (please check one)

Internet ____ Booklet ____

5. Would you be willing to read information about prostate cancer that is made available to you on the Internet? (please check one)

___ I definitely would read it on the Internet
___ I probably would read it on the Internet
___ I probably would not read it on the Internet
___ I definitely would not read it on the Internet

6. Would you be willing to participate in a research study in which you were asked to log onto an Internet website to get information about the study and to fill out questionnaires on the Internet? (please check one)

___ I definitely would participate
___ I probably would participate
___ I probably would not participate
___ I definitely would not participate

7. If you were given a $10 store coupon (e.g., Safeway, Blockbuster, Best Buy) to participate in the same research study described in #6 above, would you be willing to participate? (please check one)

___ I definitely would participate
___ I probably would participate
___ I probably would not participate
___ I definitely would not participate

8. How often do you get tested for prostate cancer? (please check one)

___I've never been tested ___Every 3-6 months ___Every year ___Every 1-2 yrs ___Less often
___Don't know/don't remember

9. Approximately how many times have you been tested for prostate cancer in your lifetime? (This includes the PSA blood test and/or rectal exam). (please check one)

___0 ___1-2 ___3-4 ___5-10 ___11+

10. Based on what you know about prostate cancer, do you think that medical experts agree that all men should be tested for prostate cancer?

____I think that experts do agree on this ____I think that experts do not agree on this ____Not sure

11. Why are you here today? (check all that apply)

___Just a check-up
___Illness
___To bring a friend/relative to a doctor's appointment
___For blood tests or other laboratory work
___Other (please specify): _________

12. How old are you? ____________

13. What is your marital status?

___Never married ___Married ___Living as married ___Separated/Divorced ___Widowed

14. How far did you go in school? (please check one)

___less than high school ___high school graduate or GED ___vocational/trade school

___some college ___college graduate ___some graduate school

___graduate/professional degree

15. To what racial/ethnic group do you belong?

___ Black/African American ___ Hispanic/Latino ___Asian/Pacific Islander

___Native American ___Caribbean or West Indian ___White ___Other: ___________

Thank you very much for completing this questionnaire. We sincerely appreciate your time.
