# Supplementary material for: The development of a web- and a print-based decision aid for prostate cancer screening
Source: BMC Med Inform Decis Mak. 2010 Mar 3;10:12. doi: 10.1186/1472-6947-10-12 (PMC2845091; doi:10.1186/1472-6947-10-12)
Supplement: Additional file 3 — Supp File 3. Pilot Screening and Internet Usage Questionnaire-Feasibility Study 2 - June 2006 [file 1472-6947-10-12-S3.PDF]

Internet Based Education for Prostate Cancer Screening

Please help us to understand the best ways to educate men about prostate cancer screening on the Internet. We appreciate all feedback. Thank you very much for your time and participation in our project.

1. Do you use a computer at home?
___yes GO TO #2
___no GO TO #8

2. What type of computer do you have at home?
___PC (e.g., Dell, Gateway, Compaq)
___Mac (Apple)
___other (please describe: ________________________________)
___not sure

3. How old is your computer in your home?
___less than 2 years old
___2-5 years old
___greater than 5 years old
___not sure

4. On your computer at home, do you have? (check all that apply)
___CD-ROM drive
___USB port (e.g, memory sticks connect to the USB port on a computer)
___Not Sure

5. Do you have an internet connection for your home computer?
___yes GO TO # 6
___no GO TO # 8

6. How do you connect to the Internet on your home computer?
___through the telephone line ("Dial-up")
___high speed connection (Broadband, DSL, cable etc.)
___other (please describe:______________________)
___not sure

7. How often do you use the Internet at home? *(please check one)*
___never or rarely
___a few times a year
___a few times per month
___once per week
___several times per week
___daily

8. Do you use a computer at work?
___yes
___no GO TO #17

9. Can you use your work computer for personal use?
___yes
___no GO TO #17

10. Do you have an Internet connection on your work computer?
____yes
____no GO TO #14

11. Can you use the Internet for personal use at work?
___yes
___no GO TO #17

12. How do you connect to the Internet on your work computer?
___through the telephone line ("Dial-up")
___high speed (Broadband, DSL, cable etc.)
___other (please describe:_____________________)
___not sure

13. How often do you use the Internet at work? *(please check one)*
___never or rarely
___a few times a year
___a few times per month
___once per week
___several times per week
___daily

14. What type of computer do you have at work?
___PC (e.g., Dell, Gateway, Compaq)
___Mac (Apple)
___other (please describe: _______________________________)
___not sure

15. On your computer at work, do you have? (check all that apply)
___CD-ROM drive
___USB port (e.g, memory sticks connect to the USB port on a computer)
___Not Sure

16. How old is your computer at work?
___less than 2 years old
___2-5 years old
___greater than 5 years old
___not sure

17. If you do not have access to a high speed Internet connection at home or work, would you be willing to go to another location (e.g., the hospital, library, friend's computer, Internet café) to access our prostate cancer screening website on the Internet?
___yes (please indicate which of the above options would be most convenient for you: ____________________)
___no
___not sure

18. If you are not comfortable accessing the Internet on your own, would you ask a friend or family member to help you use our prostate cancer screening website?
___yes
___no
___not sure

19. Would you prefer to receive health-related information on the Internet or as a booklet? *(please check one)*

Internet ____ Booklet ____

20. Why are you here today? *(check all that apply)*
___For a check-up
___Illness
___To bring a friend/relative to a doctor's appointment
___For blood tests or other laboratory work
___Other (please specify): ______________________

21. If you are here today to bring a friend/relative to a doctor's appointment, are you also a patient in this clinic?

___yes ___no ____N/A

22. How old are you? ____________

23. What is your marital status?

___Never married ___Married ___Living as married ___Separated/Divorced ___Widowed

24. How far did you go in school? *(please check one)*

___less than high school ___high school graduate or GED ___vocational/trade school ___some college

___college graduate ___some graduate school ___graduate/professional degree

25. To what racial/ethnic group do you belong?

___ Black/African American ___ Hispanic/Latino ___Asian/Pacific Islander ___Native American

___Caribbean or West Indian ___White ___Other: ____________________

Thank you very much for completing this questionnaire. We sincerely appreciate your time.
This next section asks you to look at the information that is on the back of a container of a pint of ice cream and answer a few questions about it. We are asking these questions because we are interested in the best ways to communicate medical and numerical information to people. *Participants are given a hard copy of the NVS nutrition label to hold and to which they can refer, as needed, while the interviewer asks the 6 questions out loud and records the responses.*

**Nutrition Facts**	
Serving Size	1/2 cup
Servings per container	4
Amount per serving	
Calories 250	Fat Cal 120
	%DV
**Total Fat** 13g	20%
Sat Fat 9g	40%
**Cholesterol** 28mg	12%
**Sodium** 55mg	2%
**Total Carbohydrate** 30g	12%
Dietary Fiber 2g	
Sugars 23g	
**Protein** 4g	8%

* Percent Daily Values (DV) are based on a 2,000 calorie diet. Your daily values may be higher or lower depending on your calorie needs.

Ingredients: Cream, Skim Milk, Liquid Sugar, Water, Egg Yolks, Brown Sugar, Milkfat, Peanut Oil, Sugar, Butter, Salt, Carrageenan, Vanilla Extract.

Questions to be asked by the interviewer.

READ TO SUBJECT: This information is on the back of a container of a pint of ice cream.

QUESTIONS

1. If you eat the entire container, how many calories will you eat? __________

Answer 1,000 is the only correct answer

2. If you are allowed to eat 60g of carbohydrates as a snack, how much ice cream could you have? ____________

Answer Any of the following is correct:
1 cup (or any amount up to 1 cup)
Half the container
Note: If patient answers "2 servings" ask, "How much ice cream would that be if you were to measure it into a bowl?"

3. Your doctor advises you to reduce the amount of saturated fat in your diet. You usually have 42g of saturated fat each day, which includes 1 serving of ice cream. If you stop eating ice cream, how many grams of saturated fat would you be consuming each day? _____________

Answer 33 is the only correct answer

4. If you usually eat 2500 calories in a day, what percentage of your daily value of calories will you be eating if you eat one serving? _____________

Answer 10% is the only correct answer

Pretend you are allergic to the following substances:
Penicillin, peanuts, latex gloves, and bee stings.

5. Is it safe for you to eat this ice cream? Yes ________ No_________ DK __________

Answer No

6. (Ask only if patient responds "no" to question 5): Why not?________________

Answer Because it has peanut oil.

Weiss, D. et al., (2005). Quick Assessment of Literacy in Primary Care: The Newest Vital Sign. Annals of Family Medicine, 3(6): 514-522.
