# Supplementary material for: The development of a web- and a print-based decision aid for prostate cancer screening
Source: BMC Med Inform Decis Mak. 2010 Mar 3;10:12. doi: 10.1186/1472-6947-10-12 (PMC2845091; doi:10.1186/1472-6947-10-12)
Supplement: Additional file 4 — Supp File 4. Booklet Usability Testing Questionnaire [file 1472-6947-10-12-S4.PDF]

Prostate Cancer Screening: Fostering Informed Decisions

We thank you for your participation today. We would appreciate it if you took a moment to answer this questionnaire for us. There are two parts to this questionnaire. The first part asks for your feedback about the educational booklet, and the second part asks questions about you and your health. Please take your time in answering all of the questions. Your responses will be kept confidential.

Part I: Questions about the booklet

1. How would you rate the amount of information in the booklet?
___much less information than was needed
___a little less information than was needed
___about the right amount of information
___a little more information than was needed
___a lot more information than was needed

2. How would you rate the length of the booklet?
___much too long
___a little too long
___just about right
___should have been a little longer
___should have been much longer

3. How clear was the information in the booklet?
___everything was clear
___most things were clear
___some things were clear
___many things were unclear

4. What did you think that the overall message of the **booklet** was suggesting?
___ that men should definitely not get screened
___ that men should probably not get screened
___ it did not suggest one decision or the other
___ that men should probably get screened
___ that men should definitely get screened

5. Did the booklet make you think of new questions to ask your doctor? ___yes ___no

6. Did the booklet help you explore the benefits and limitations of screening?
____ helped very much to explore the benefits and limitations
____ helped somewhat to explore the benefits and limitations
____ helped a little to understand the benefits and limitations
____ was not helpful

7. Did the information that was presented in the booklet address your questions about prostate cancer and prostate cancer screening?

___Yes, it completely addressed my questions
___Yes, it addressed most of my questions
___It addressed some of my questions
___No, it did not adequately address my questions

8. Did the booklet make you feel nervous or fearful about prostate cancer screening?

___Yes, it made me nervous
___Yes, it made me somewhat nervous
___It made me a little nervous
___No, it did not make me nervous

9. What else would you have liked to see in this booklet?

10. What would you like to see removed from this booklet?

Part II: Questions about You and Your Health

1. What is your age?   ________

2. What is your marital status?
____never married
____married
____living in a marriage-like relationship
____separated
____divorced
____widowed
____other: ________

3. How many years of school have you completed?
____8th grade or less
____some high school
____high school graduate or GED
____some college
____college graduate
____graduate work/degree

4. Are you currently employed?
____not employed
____retired
____full-time employed
____part-time employed

5. To which ethnic group do you belong?
____White
____Black or African-American
____Hispanic or Latino
____Caribbean or West Indian
____Asian
____Native Hawaiian or Other Pacific Islander
____Other: _______________

6. Do you have health insurance? ______yes ______no

7. How many times have you been screened for prostate cancer?

______number of PSA blood tests
______number of DRE tests

8. How often do you get screened for prostate cancer?
_____every 3-6 months
_____annually
_____every 2 years
_____don't know

9. What was the approximate date of your most recent examination for prostate cancer?
_____/______/_______

10. Have you ever had an abnormal screening result from a prostate cancer examination?
______yes ______no

11. Have you ever been diagnosed with prostate cancer?
______yes ______no

12. Please think about how good or bad your health is, on a 100 point scale. Zero is the equivalent of the worst health you can imagine, and 100 is equivalent to the best health you can imagine. Where would you put yourself on this scale, in terms of your own current health?

-…………………………………………-…………………………………………-
0 50 100

Your rating: ______________
